# A study of mobile learning for higher education students in Guangzhou

**DOI:** 10.1186/2193-1801-3-S1-P3

**Published:** 2014-12-04

**Authors:** Hugo Chun-hung Wong

**Affiliations:** Computing & Mathematics Section, Youth College (Yeo Chei Man), Kragujevac, Hong Kong

## Background

Recently, ‘mobile learning’ (M-learning) has become a popular research topic in investigating how to use mobile device for teaching and learning. M-learning is a mode of learning using smartphones, phablets, tablets, netbooks or notebooks as the learning device and is a part of electronic learning (e-learning). However, M-learning is more flexible than e-learning [1, 2] because students can learn anywhere, anytime. Moreover, M-learning is an important new learning strategy because mobile devices are now necessary tools in students’ daily life. As noted by Yousuf [3], M-learning is ‘more interactive, involving more contact, communication, and collaboration with people’. M-learning is a student-oriented approach that makes learning informal, independent and collaborative[3].

Based on the theoretical model Framework for the Rational Analysis of Mobile Education (FRAME) [4], this study examined the practices and attitudes relating to M-learning for university students’ studying in Guangzhou, particularly to examine what and how knowledge will be acquired using mobile devices in learning. This model is valuable in different aspects as it can be used:

- as an indicator for the development of future mobile devices;
- for the development of learning materials; and
- for the design of teaching and learning strategies for mobile education.

## Methods

Mixed-method research design is a procedure for collecting, analyzing, and ‘mixing’ both quantitative and qualitative research and is a single study method to understand the research problem [5]. Because the purpose of this study was to draw a full picture to understand the practices of using M-learning for Guangzhou’s university students, a mixed-method research strategy was adopted and implemented.

To collect the quantitative data, a set of tailor-made questionnaires was distributed to 495 Guangzhou university students in March 2013. The objective of the questionnaire was to collect quantitative data on the practices and attitudes of university students in relation to M-learning. As qualitative data, a pre-survey focus group interview, diary records and post-survey individual interviews were completed from April to June of 2013. The aim of the pre-survey focus group interview for identifying and sharing the use of mobile devices was to promote learning with Guangzhou’s university students. The diary record provided a means to record students’ mobile learning practices, and the post-survey interview provided opportunities for the participants to express their views and feelings. Therefore, the researcher could easily follow up on unexpected results or more deeply examine the motivation of the respondents and their reasons for responding as they did.

## Results and conclusions

After the quantitative data and qualitative information were collected, the qualitative information was quantified, and matrix tables were built to compare and contrast the quantitative variables and qualitative themes with text data quotes. Thus, to facilitate informal learning in Guangzhou’s institutions of higher education, this study provided in-depth analysis and clearly explained the practices and purposes of using mobile devices.

This study found that Guangzhou’s university students prefer to use mobile devices for learning outside rather than in the classroom. In addition, assessment methods were crucial factors for using mobile device to learn. Lastly, reading and searching were the most performed of the M-learning activities. These findings can assist teachers to design M-learning activities either for classroom or out-of-classroom learning and motivate students’ learning attitudes and practices.
